# Neurological outcomes and survival after prehospital ECPR: the impact of low-flow time

**DOI:** 10.3389/fcvm.2026.1853927

**Published:** 2026-06-12

**Authors:** Zishu Xu, Zhijing Xu, Shiqiong Su, Ping Ren, Linkai Huang, Lu Qi

**Affiliations:** Department of Intensive Care Medicine, The Third People’s Hospital of Henan Province, Zhengzhou, China

**Keywords:** extracorporeal cardiopulmonary resuscitation, favorable neurological outcome, low-flow time, out-of-Hospital cardiac arrest, survival

## Abstract

**Objective:**

To investigate the association between low-flow time (LFT) and outcomes in out-of-hospital cardiac arrest (OHCA) patients treated with prehospital extracorporeal cardiopulmonary resuscitation (ECPR).

**Methods:**

This retrospective study included OHCA patients receiving prehospital ECPR (July 2023–August 2024). LFT, defined as the interval from conventional CPR start to ECPR flow initiation, was analyzed continuously (per 10 min increment). Due to limited events (13 favorable neurological outcomes), the primary logistic regression adjusted for two prespecified confounders (age, initial shockable rhythm). A sensitivity analysis was performed in patients with witnessed arrest and bystander CPR (no-flow time of approximately 0). All analyses are exploratory.

**Results:**

Among 76 patients (mean age 58.80 ± 14.84 years, mean LFT 60.33 ± 13.89 min), survival to hospital discharge was 34.2% (26/76) and favorable neurological outcome 17.1% (13/76). Each 10 min LFT increase was associated with lower survival (aOR 0.557; 95% CI 0.368–0.844; *P* = 0.006) and favorable neurological outcome (aOR 0.461; 95% CI 0.255–0.834; *P* = 0.011). In the sensitivity subgroup (witnessed + bystander CPR, *n* = 44, 9 favorable outcomes), the univariable OR for favorable outcome was 0.395 (95% CI 0.176–0.886; *P* = 0.024), consistent with the primary estimate. Exploratory ROC analysis for favorable neurological outcome gave an AUC of 0.750 (95% CI 0.603–0.896), but the derived cutoff (55.5 min) is not proposed for clinical use.

**Conclusions:**

In this single-center study, longer LFT (per 10 min) was associated with worse outcomes, consistent in a no-flow-time-controlled subgroup. Given the exploratory design, external validation is required. No definitive LFT threshold can be recommended.

## 1 Introduction

Out-of-hospital cardiac arrest (OHCA) remains a critical global public health challenge with high mortality. In developed countries, the survival rate for OHCA ranges from 10% to 30% (1). In contrast, a multicenter study from China indicated a considerably lower survival to hospital discharge rate of 1.15%, with only 0.83% of patients achieving a favorable neurological outcome (2). This significant disparity underscores the urgent need for more effective interventions to improve patient prognosis. Conventional cardiopulmonary resuscitation (CCPR) can generate only 25%–30% of normal cardiac output (3), and the duration of this low-flow state is strongly associated with the risk of end-organ damage. Extracorporeal cardiopulmonary resuscitation (ECPR), which utilizes veno-arterial extracorporeal membrane oxygenation (VA-ECMO), provides more efficient gas exchange and organ perfusion, thereby serving as a bridge to definitive therapy (4). Its hemodynamic benefits may help mitigate irreversible brain injury (5). Evidence suggests that shorter low-flow time (LFT) is associated with improved survival and neurological outcomes in patients treated with ECPR (6, 7). However, existing studies have primarily focused on in-hospital ECPR, and evidence regarding time-sensitive thresholds in pre-hospital settings remains limited. Prehospital ECPR has the potential to minimize LFT by circumventing transport-related delays (8), yet its implementation poses significant environmental and technical challenges.

This retrospective study analyzed data from OHCA patients treated with prehospital ECPR. The study aimed to investigate the association between LFT and clinical outcomes in a Chinese prehospital ECPR cohort, with the goal of providing descriptive evidence to optimize resuscitation strategies. Rather than proposing a novel threshold, our exploratory findings are positioned within the context of previously reported LFT ranges (e.g., Otani et al. 58 min) (7).

## 2 Materials and methods

### 2.1 Study design and participants

This single-center, retrospective cohort study included adult patients with OHCA who received prehospital ECPR and were subsequently admitted to the Department of Intensive Care Medicine of the Third People's Hospital of Henan Province between July 2023 and August 2024. The exclusion criteria were as follows: patients who received in-hospital ECPR; age ≤18 years; termination of treatment due to family request; or incomplete key clinical data, including missing patient outcomes or LFT. The study was conducted in accordance with the principles of the Declaration of Helsinki and received ethical approval from the Ethics Committee of the Third People's Hospital of Henan Province (Reference Number: 2025SZSYLCYJ0703). Written informed consent for the ECPR procedure was obtained from a family member before cannulation; the requirement for informed consent for retrospective data analysis was waived by the Ethics Committee.

### 2.2 Prehospital ECPR implementation

**Figure 1:**
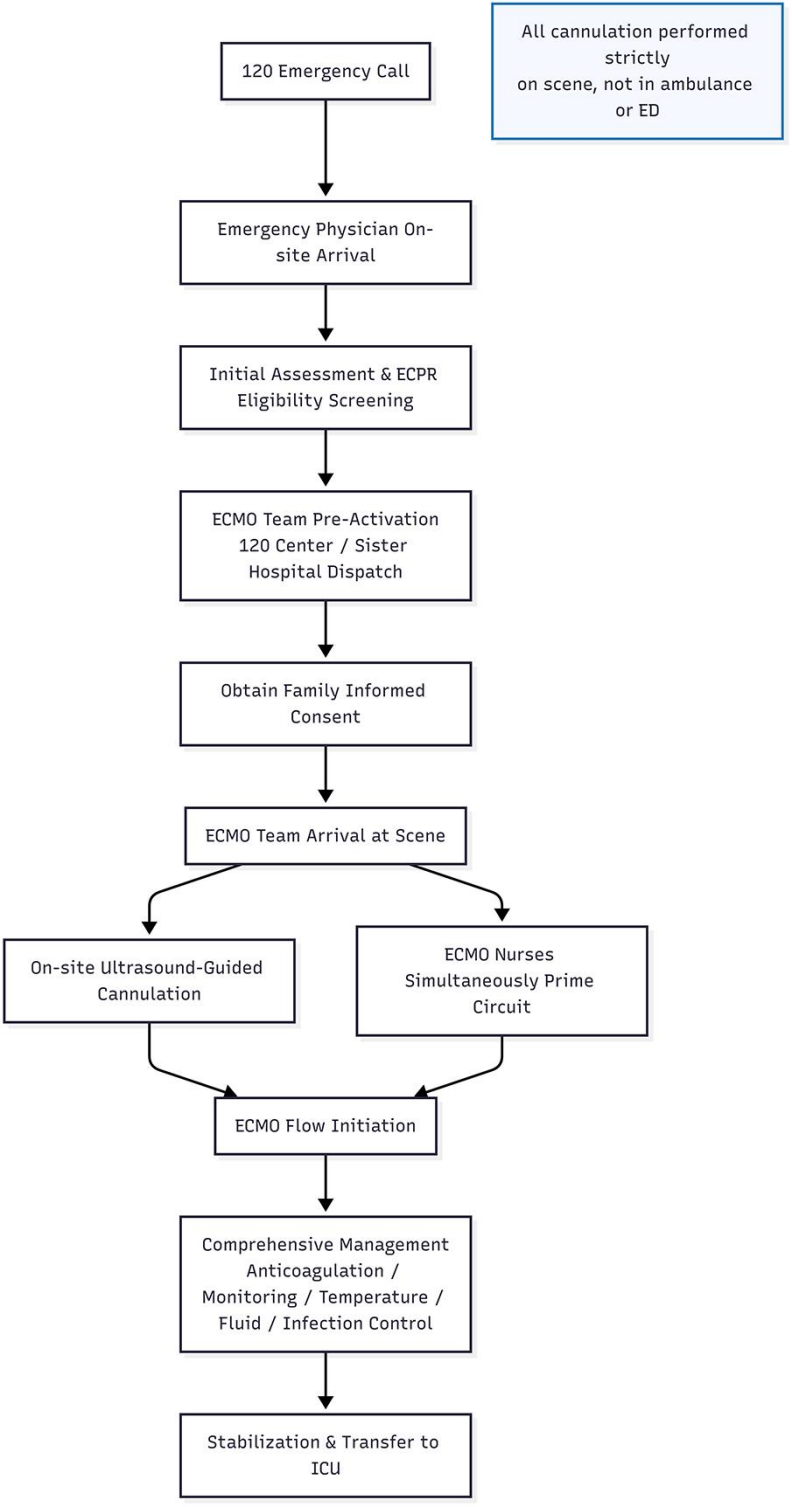
Qualitative flow diagram of prehospital ECPR implementation process.

The emergency physician conducts an on-site initial assessment to determine the patient's eligibility for ECPR and pre-activates the ECMO team. Upon pre-activation, the ECMO team (two critical care physicians, two ECMO specialist nurses) is dispatched from our tertiary hospital and travels to the scene by a dedicated emergency vehicle equipped with a portable ECMO system. All prehospital extracorporeal cardiopulmonary resuscitation (ECPR) cannulation procedures are performed at the emergency scene, rather than in an ambulance or emergency department. After obtaining informed consent from the family, two critical care physicians perform ultrasound-guided cannulation (15–17 Fr arterial, 21–25 Fr venous). Simultaneously, two ECMO specialist nurses rapidly prime the ECMO circuit, assist during cannulation, and manage subsequent aspects including circuit maintenance, anticoagulation therapy, flow adjustment, temperature control, hemodynamic monitoring, fluid management, and infection prevention measures. The patient is then transported to the cardiac catheterization laboratory or ICU under ongoing ECMO support. A qualitative flow diagram is provided to illustrate the above prehospital ECPR implementation process (Figure 1).

#### 2.2.1 Geographic coverage and transport distances

The catchment radius from our tertiary center is approximately 20 km. Based on available GPS data from 40 of 76 cases (consecutive cases with retrievable ambulance GPS records; no other selection criteria were applied), transport distances from scene to hospital ranged from 5 to 25 km. The median transport distance could not be reliably calculated due to incomplete records.

Given the retrospective study design, the overall median time from pre-activation to ECMO team arrival on scene could not be determined for the entire cohort. A representative case with complete timestamp data is presented in Supplementary Table S1, showing sequential time intervals from collapse to hospital arrival, including the time from prehospital activation to scene arrival and from on-site cannulation to successful ECMO flow initiation.

### 2.3 Data collection

The collected case data include: gender, age, medical history, APACHE II score, cause of cardiac arrest (cardiogenic, non-cardiogenic, etc.), witnessed cardiac arrest, bystander cardiopulmonary resuscitation (CPR), initial cardiac rhythm (shockable, non-shockable), brief return of spontaneous circulation (ROSC) before ECMO initiation, time of ECMO establishment, LFT, survival status at hospital discharge, and cerebral performance category at hospital discharge. LFT is defined as the time from the start of CCPR to the establishment of prehospital ECPR (9). “Time of ECMO establishment” is defined as the interval from the start of ultrasound-guided femoral vessel cannulation to the successful initiation of ECMO flow (cannulation-to-flow time).

### 2.4 Outcomes

The primary outcome was survival to hospital discharge. The secondary outcome was favorable neurological outcome, defined as a cerebral performance category (CPC) of 1 or 2 at hospital discharge.

### 2.5 Statistical analysis

Continuous variables are presented as mean ± SD or median (IQR); categorical as *n* (%). No imputation was performed for missing covariate data; only complete cases were analyzed. LFT was analyzed as a continuous variable (per 10 min). Due to limited events (13 favorable neurological outcomes), the primary logistic regression adjusted for only two confounders (age, initial shockable rhythm). All analyses are exploratory. To address unmeasured no-flow time, a sensitivity analysis was performed in patients with witnessed arrest and bystander CPR (no-flow time of approximately 0) using univariable logistic regression. Exploratory ROC analysis reported AUC but did not propose a clinical cutoff. Significance was set at *P* < 0.05. SPSS 26.0 was used.

## 3 Results

### 3.1 Clinical data

**Figure 2:**
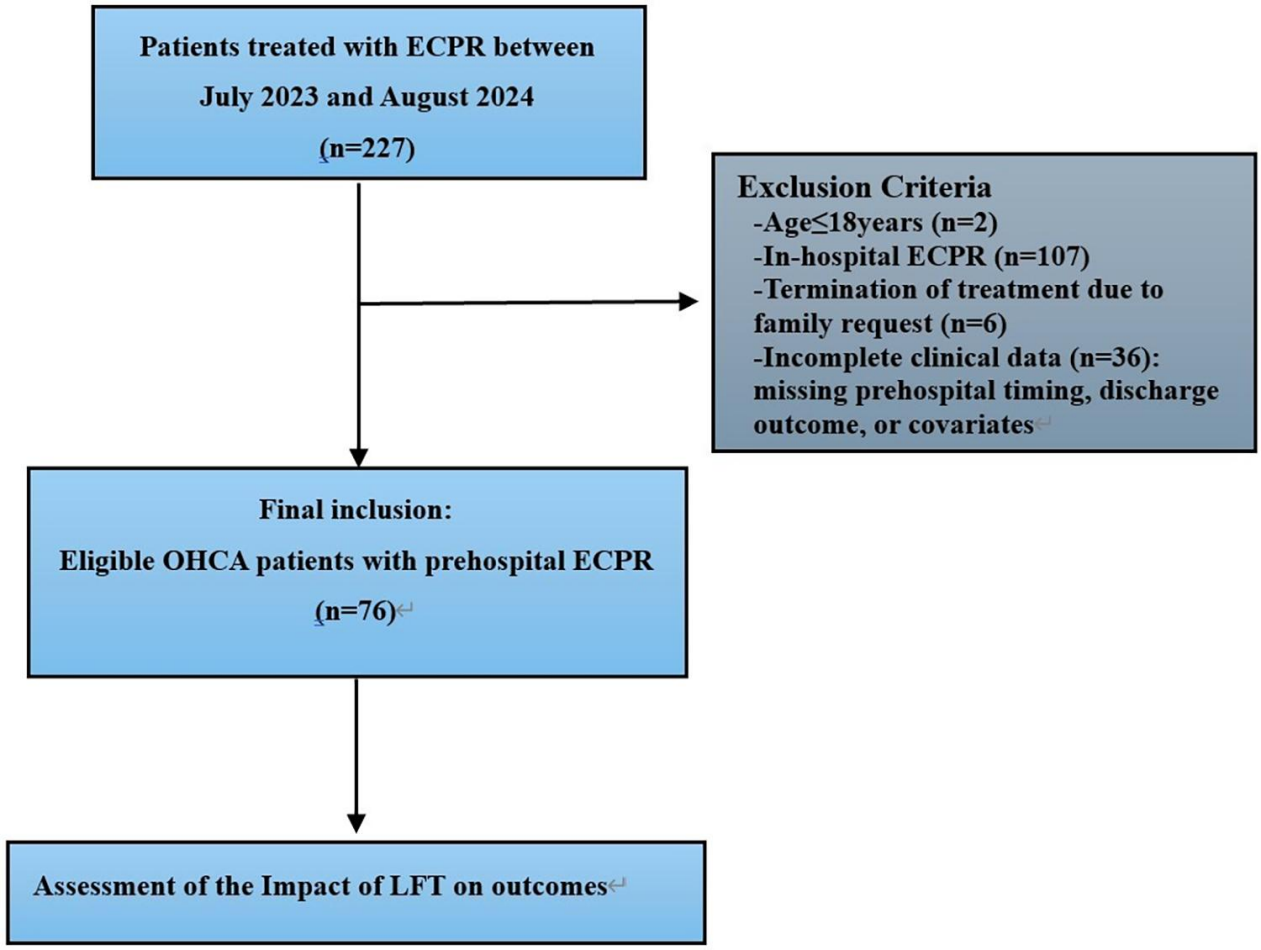
Study flow chart showing selection process of the study cohort. ECPR: extracorporeal cardiopulmonary resuscitation.

From July 2023 to August 2024, 227 consecutive patients were treated with ECPR in the Department of Intensive Care Medicine of the Third People's Hospital of Henan Province. After excluding 107 in-hospital ECPR cases, 2 patients aged <18 years, and 6 patients in whom family withdrew treatment, 112 OHCA patients were potentially eligible. Of these, 36 (32.1%) were excluded because of incomplete clinical data (missing prehospital timing records, discharge outcome status, or baseline covariates). No further clinical information could be retrieved for these 36 patients, precluding direct comparison with the included cohort. The remaining 76 adult OHCA patients treated with prehospital ECPR were included in the analysis (Figure 2). No patient was transferred to another center post-ECMO, and all discharge outcomes were determined at our hospital.

The baseline characteristics of the patients were presented in Table 1. Of the 76 patients, 77.6% (59/76) were male, with a mean age of 58.80 ± 14.84 years and a mean LFT of 60.33 ± 13.89 min. The overall survival rate to hospital discharge was 34.2% (26/76), and the rate of favorable neurological outcome was 17.1% (13/76).

**Table 1:** Baseline characteristics of the entire study cohort.

Characteristics	Value(*N* = 76)
Demographic and clinical characteristics	
Male, *n* (%)	59 (77.6)
Age(years), mean ± SD	58.80 ± 14.84
APACHE II score(points), mean ± SD	29.11 ± 6.53
Cardiovascular history, *n* (%)	20 (26.3)
Cause of cardiac arrest, *n* (%)	
Cardiogenic	47 (61.8)
Non-cardiogenic	14 (18.4)
Unexplained	15 (19.7)
Witnessed cardiac arrest, *n* (%)	59 (77.6)
bystander CPR, *n* (%)	53 (69.7)
Initial shockable rhythm, *n* (%)	15 (19.7)
Brief ROSC before ECMO initiation,n(%)	28 (36.8)
LFT(min), mean ± SD	60.33 ± 13.89
Time of ECMO establishment (min), mean ± SD	20.11 ± 6.99
Outcomes	
survival to hospital discharge, *n* (%)	26 (34.2)
favorable neurological outcome (CPC 1–2), *n* (%)	13 (17.1)

Values for continuous variables are expressed as mean ± standard deviation; counts (percentages) are used for categorical variables. Time of ECMO establishment: time from start of ultrasound guided cannulation to ECMO flow initiation. SD, standard deviation; CPC, Cerebral Performance Category; APACHE II, Acute Physiology and Chronic Health Evaluation II; CPR, cardiopulmonary resuscitation; ECMO, extracorporeal membrane oxygenation; ROSC, return of spontaneous circulation; LFT, low-flow time.

### 3.2 Association between LFT and outcomes in prehospital ECPR patients

LFT was analyzed as a continuous variable per 10 min increment. Due to the limited number of favorable neurological outcomes (*n* = 13), the primary logistic regression model adjusted for only two prespecified confounders: age and initial shockable rhythm.

Survival to hospital discharge (Table 2): Each 10 min increase in LFT was associated with significantly lower odds of survival to discharge in both univariable analysis (OR 0.557; 95% CI 0.370–0.838; *P* = 0.005) and after adjustment for age and initial shockable rhythm (aOR 0.557; 95% CI 0.368–0.844; *P* = 0.006).

**Table 2:** Association between low flow time (per 10 min increment) and outcomes.

Outcome	Model	OR/aOR	95% CI	*P* value
Survival to hospital discharge	Univariable	0.557	0.370–0.838	0.005
	Multivariable^a^	0.557	0.368–0.844	0.006
Favorable neurological outcome	Univariable	0.466	0.264–0.824	0.009
	Multivariable^a^	0.461	0.255–0.834	0.011

a Adjusted for age and initial shockable rhythm.

All analyses are exploratory due to the limited sample size and number of events.

Favorable neurological outcome (Table 2): Similarly, each 10 min increase in LFT was associated with lower odds of favorable neurological outcome in univariable analysis (OR 0.466; 95% CI 0.264–0.824; *P* = 0.009) and after adjustment (aOR 0.461; 95% CI 0.255–0.834; *P* = 0.011).

Sensitivity analysis for no-flow time: To address potential residual confounding by unmeasured no-flow time (interval from collapse to first compression), we restricted the analysis to patients with witnessed arrest and bystander CPR (*n* = 44), in whom no-flow time is approximately zero. In this subgroup, 19 patients (43.2%) survived to discharge and 9 (20.5%) achieved favorable neurological outcome. Univariable logistic regression showed that each 10 min increase in LFT remained associated with worse outcomes: the OR for survival to hospital discharge was 0.519 (95% CI 0.304–0.888; *P* = 0.017) and the OR for favorable neurological outcome was 0.395 (95% CI 0.176–0.886; *P* = 0.024). These estimates are consistent with the primary analysis, suggesting that residual confounding by no-flow time is unlikely to explain the observed association.

### 3.3 Exploratory ROC analysis

**Figure 3:**
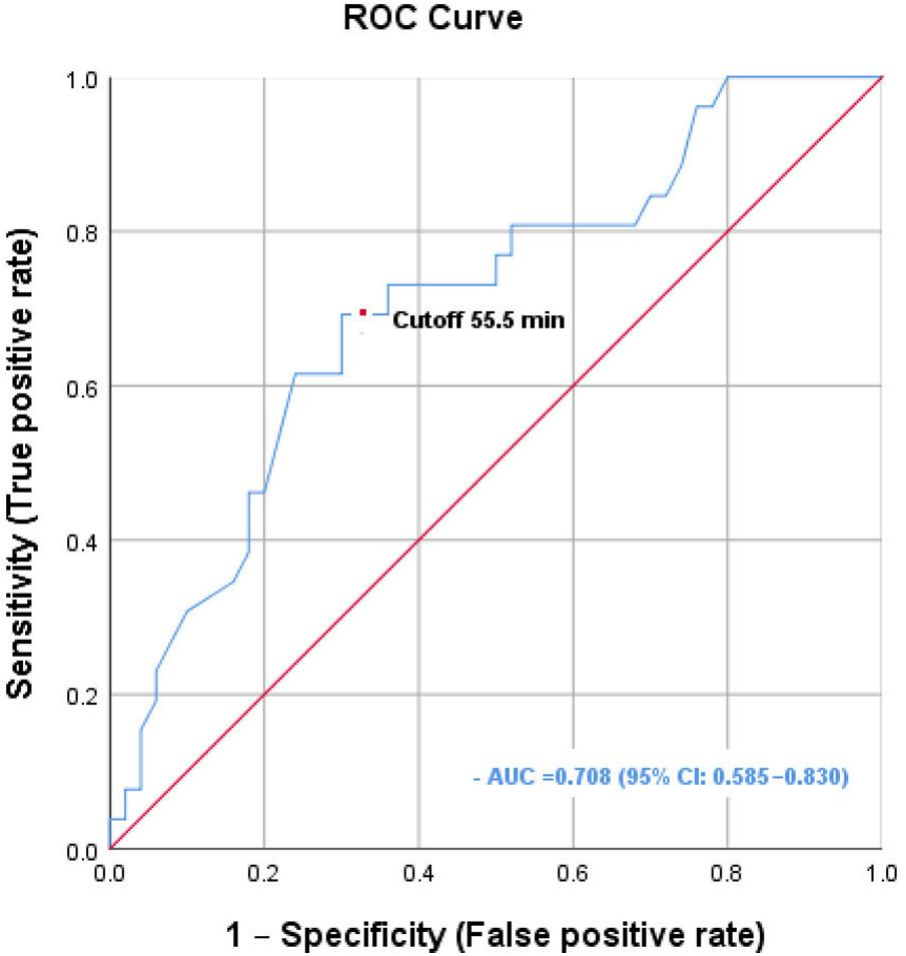
Shows the predictive value of LFT for survival to hospital discharge in prehospital ECPR patients.

**Figure 4:**
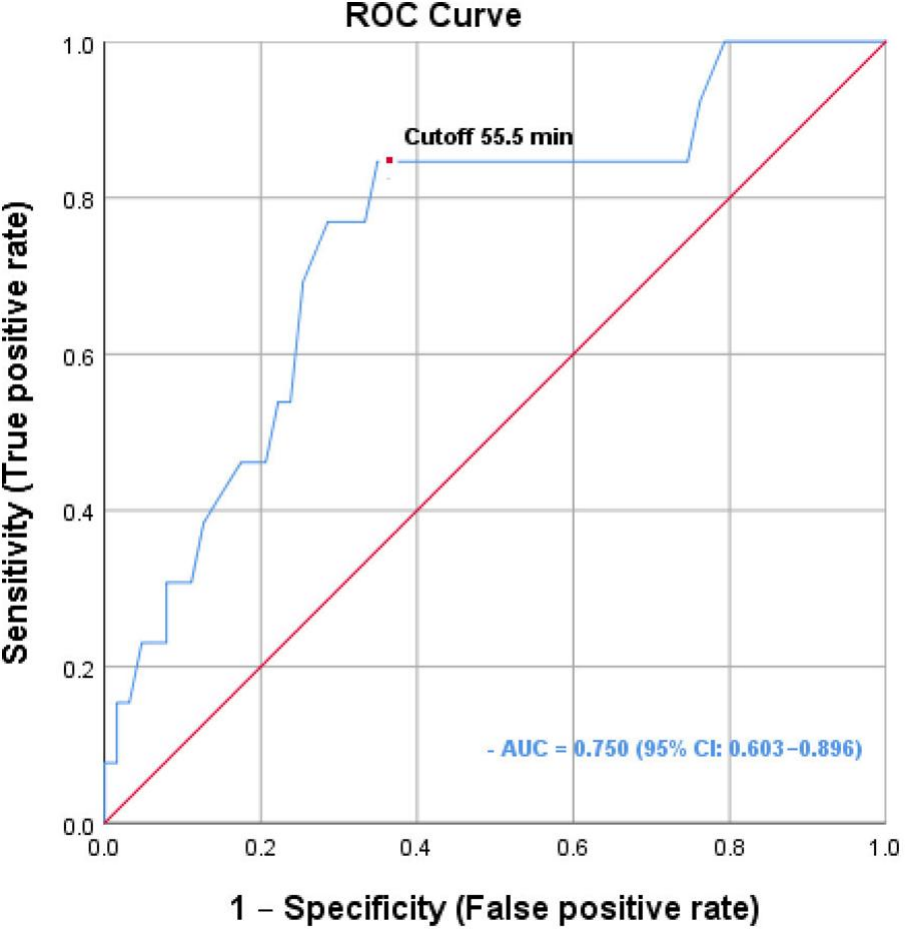
Shows the predictive value of LFT for favorable neurological outcomes in prehospital ECPR patients.

We performed exploratory receiver operating characteristic (ROC) analyses to assess the discriminative ability of LFT for outcomes. Figure 3 shows the ROC curve for survival to hospital discharge. The area under the curve (AUC) was 0.708 (95% CI: 0.585–0.830). The analysis yielded a cutoff value of 55.5 min (sensitivity 70%, specificity 69.2%). Figure 4 shows the ROC curve for favorable neurological outcome. The AUC was 0.750 (95% CI: 0.603–0.896). The wide confidence interval indicates substantial uncertainty in the discriminative ability. The same cutoff value of 55.5 min was identified (sensitivity 84.6%, specificity 65.1%). Because cutoffs derived from small samples are unstable and tend to overperform, these data-driven thresholds are not proposed for clinical use and require external validation.

## 4 Discussion

This study provides a comprehensive analysis of the association of low-flow time (LFT) with clinical outcomes in adult OHCA patients treated with prehospital ECPR. Our principal findings demonstrate that longer LFT was associated with reduced survival to hospital discharge (aOR per 10 min increase = 0.557, 95% CI 0.368–0.844) and favorable neurological outcome (aOR per 10 min increase = 0.461, 95% CI 0.255–0.834), after adjustment for age and initial shockable rhythm. Univariable analyses yielded similar estimates (for survival to hospital discharge: OR 0.557; for favorable neurological outcome: OR 0.466). Given the limited sample size and number of events, all analyses are exploratory.

To address potential residual confounding from unmeasured no-flow time, we performed a sensitivity analysis restricted to patients with witnessed arrest and bystander CPR (*n* = 44, no flow time of approximately 0). In this subgroup, each 10 min increase in LFT remained associated with worse outcomes (OR for favorable neurological outcome 0.395, 95% CI 0.176–0.886). This finding was consistent with the primary estimate and supports the robustness of our results. This argues against substantial confounding by no-flow time, although any residual bias would likely lead to overestimation of LFT's effect.

Our results are consistent with existing literature but provide exploratory information on a possible threshold. The ROC analyses showed an AUC of 0.750 (95% CI 0.603–0.896) for favorable neurological outcome and identified a cutoff of 55.5 min with sensitivity 84.6% and specificity 65.1%. However, because cutoffs derived from small samples are unstable and tend to overperform, this data-driven threshold is not proposed for clinical use and requires external validation. The higher discriminatory power for neurological outcomes (AUC 0.750) is particularly noteworthy, suggesting that LFT may be most valuable in predicting quality-of-survival endpoints rather than mere survival itself, reflecting the neural tissue's heightened sensitivity to ischemic injury.

These findings align with the established pathophysiological understanding that extended periods of inadequate perfusion exacerbate ischemic injury to vital organs, particularly the brain and heart. ECPR, as an advanced resuscitation technique, provides continuous circulatory support for OHCA patients and mitigates end-organ damage secondary to prolonged LFT. However, its efficacy remains limited by the duration of LFT (10–12). The observed discrepancy in outcomes between OHCA and IHCA patients may be largely attributed to the substantially shorter LFT typically achieved in in-hospital settings (13).

Our data corroborate international evidence regarding LFT-related prognostic thresholds. The Japanese multicenter study confirmed superior outcomes with LFT <40 min (14), while Chen et al. demonstrated a stepwise survival decline (approximately 50% at 30 min, 30% at 60 min, and 10% at 90 min) in a mixed cardiac arrest cohort predominantly consisting of in-hospital cases (15). Another study reported that for every 10 min increase in LFT beyond 30 min, survival decreases by 25% (16). Our exploratory analysis also identified a cutoff around 55.5 min, which falls within the range proposed by Otani et al. (58 min) (7), but validation in larger cohorts is needed. Importantly, the major novelty of the present study lies not in proposing a new LFT threshold, but in describing outcomes from a real-world Chinese prehospital ECPR program, a setting less well-characterized in current global literature. Within this unique emergency care framework, our findings support that prolonged LFT remains an adverse prognostic factor for both survival and neurological recovery, reinforcing its clinical relevance for Chinese prehospital OHCA management.

The clinical imperative to minimize LFT has driven the evolution of ECPR from in-hospital to prehospital application. Bougouin et al. (11) demonstrated that prehospital ECPR was independently associated with higher survival to hospital discharge (OR = 2.9, 95% CI: 1.5–5.9, *p* = 0.002) and favorable neurological outcome (OR = 2.9, 95% CI: 1.3–6.4, *p* = 0.008). Singer et al. (5) reported 15% survival to discharge in their prehospital ECPR cohort, while Leroux's systematic review indicated that 25% of patients achieved neurologically intact survival following prehospital ECPR, with shorter LFT consistently associated with improved outcomes (17). In the present study, the observed survival to discharge rate of 34.2% is within the range of some previous international reports, representing real-world outcomes from a structured Chinese prehospital ECPR program, although we cannot exclude that this rate may be overestimated because 36 eligible patients were excluded due to incomplete data (see Limitations). Although favorable neurological outcome rates remain an area for improvement compared to pooled estimates from systematic reviews, these findings suggest the potential of standardized prehospital ECMO pathways to improve resuscitation efficacy, particularly for survival, and further support LFT as a prognostic factor within the Chinese emergency care context.

While no definitive consensus exists on the optimal LFT threshold for initiating ECPR in out-of-hospital cardiac arrest patients, the 2021 European Resuscitation Council (ERC) guidelines recommend considering ECPR when the time to establish extracorporeal support is less than 60 min from the start of CPR (18). The latest 2025 ERC guidelines further refine this window, proposing an ideal LFT range of 45–60 min for ECPR eligibility (19). Our exploratory analysis suggested a cutoff of ≤55.5 min for improved neurological recovery, which falls within this internationally recommended time frame; however, this data-driven finding is not proposed for clinical use and requires external validation in larger cohorts. Implementing such time-based selection criteria demands a well-integrated emergency care system with rapid ECMO deployment, as demonstrated by the real-world Chinese prehospital ECPR program evaluated in the present study.

## 5 Limitations

This study has several limitations. First, the single-center, retrospective design with a small sample size (76 patients, 13 favorable neurological outcomes) limits generalizability and statistical power. Findings may not apply to settings with different EMS structures, transport logistics, or lower ECPR volumes. Second, complete prehospital timestamps (e.g., ECMO team dispatch-to-arrival intervals) and transport distances (available for only 40/76 cases via GPS) were not available for all patients, precluding quantitative workflow analysis. Third, 36 of 112 eligible OHCA patients (32.1%) were excluded due to incomplete data; we could not retrieve their outcomes. If non-survivors were more likely to have missing records, the reported survival (34.2%) might be overestimated. Fourth, despite adjusting for initial shockable rhythm, the small sample size prevents complete separation of the effects of LFT and initial rhythm. Fifth, clinically relevant variables (scene-to-hospital arrival, TTM initiation/completion, prehospital adrenaline, gasping) were not collected, representing unmeasured confounders. Sixth, the ROC-derived cutoff (55.5 min) was not internally validated (e.g., via bootstrap). Therefore, the reported AUC and cutoff may be overoptimistic and require external validation in independent cohorts. Seventh, we only assessed discharge outcomes without long-term follow-up, and selection bias may exist because only ECPR-treated patients were included. Prospective multicenter studies are needed to validate our findings.

## 6 Conclusion

In this exploratory single-center study, longer low-flow time (per 10 min increment) was associated with worse outcomes after prehospital ECPR, even after adjustment for confounders and in a sensitivity analysis for no-flow time. Given the small sample size, these findings require external validation, and no definitive LFT threshold can be recommended for clinical use.

## Data Availability

The datasets presented in this article are not readily available because the dataset is not publicly accessible due to ethical and privacy restrictions. Access is granted upon reasonable request and subject to a data use agreement that prevents re-identification and limits use to approved research. Requests to access the datasets should be directed to the corresponding author directly via email. No public website listing is available for dataset requests.
