# Supplementary material for: Neurological outcomes and survival after prehospital ECPR: the impact of low-flow time
Source: Front Cardiovasc Med. 2026 Jun 12;13:1853927. doi: 10.3389/fcvm.2026.1853927 (PMC13303693; doi:10.3389/fcvm.2026.1853927)
Supplement: Supplementary file 1 [file Datasheet1.pdf]

Table S1. Representative case timeline of prehospital ECPR

Event	Cumulative time (min)	Interval (min)	Notes
Emergency call (to 120)	0	-	Witnessed collapse
EMS arrival on scene	5	5	
Start of conventional CPR (CCPR)	5	0	LFT start time
Pre activation of ECMO team (by on scene emergency physician)	15	10	Via 120
ECMO team arrival on scene	26	11	
Start of ultrasound guided cannulation	29	3	
Successful ECMO flow initiation	45	16	LFT end time
Hospital arrival (ICU/cath lab)	59	14	

Calculated low flow time (LFT) = CCPR start to ECMO flow =45-5 = 40 minutes.
